# Targeting protein kinase CK2 and CDK4/6 pathways with a multi-kinase inhibitor ON108110 suppresses pro-survival signaling and growth in mantle cell lymphoma and T-acute lymphoblastic leukemia

**DOI:** 10.18632/oncotarget.26514

**Published:** 2018-12-28

**Authors:** Amol Padgaonkar, Olga Rechkoblit, Rodgrigo Vasquez-Del Carpio, Venkat Pallela, DRC Venkata Subbaiah, Stephen C. Cosenza, Stacey J. Baker, M.V. Ramana Reddy, Aneel Aggarwal, E. Premkumar Reddy

**Affiliations:** ^1^ Department of Oncological Sciences, Icahn School of Medicine, New York 10029, NY, USA; ^2^ Department of Pharmacological Sciences, Icahn School of Medicine, New York 10029, NY, USA; ^3^ Present Address: Prescient Healthcare Group, Jersey City 07302, NJ, USA; ^4^ Present address: Sandoz, a Novartis Company, Miami 33126, FL, USA; ^5^ Present address: Pfizer, Collegeville 19426, PA, USA; ^6^ Present address: Carnegie Pharmaceuticals, Monmouth Junction 08852, NJ, USA

**Keywords:** mantle cell lumphoma, T-cell acute lymphoblastic leukemia, CDK4, CK2

## Abstract

Overexpression and constitutive activation of CYCLIN D1 and Casein Kinase 2 are common features of many hematologic malignancies, including mantle cell lymphoma (MCL) and leukemias such as T-cell acute lymphoblastic leukemia (T-ALL). Although both CK2 and CDK4 inhibitors have shown promising results against these tumor types, none of these agents have achieved objective responses in the clinic as monotherapies. Because both proteins play key roles in these and other hematological malignancies, we have analyzed the therapeutic potential of ON108110, a novel dual specificity ATP-competitive inhibitor of protein kinase CK2 as well as CDK4/6 in MCL and T-ALL. We show that in cell growth inhibition assays, MCL and T-ALL cell lines exhibited increased sensitivity to ON108110 when compared to other tumor types. Treatment with ON108110 reduced the level of phosphorylated RB-family proteins. In addition, ON108110 treatment resulted in concentration dependent inhibition of PTEN phosphorylation and a concomitant decrease in PI3K-AKT signaling mediated by CK2. Accordingly, cells treated with ON108110 rapidly accumulated in the G_0_/G_1_ stage of the cell cycle as a function of increasing concentration followed by rapid onset of apoptosis. Together, these results indicate that dual inhibition of CK2 and CDK4/6 may be an efficient treatment of MCL and T-ALLs displaying upregulation of CK2/PI3K and CDK4 signaling pathways.

## INTRODUCTION

Mantle cell lymphoma (MCL) is a well-defined and aggressive B-cell non-Hodgkin's lymphoma (B-NHL) that is genetically characterized by a t(11;14)(q13;q32) chromosomal translocation which results in constitutive over-expression of CYCLIN D1 [1–3]. CYCLIN D1 over-expression is considered to be the hallmark of MCL, and is closely correlated with the proliferative rate of these malignant cells [4, 5]. During cell cycle progression, CYCLIN D1 complexes with its catalytic partners, cyclin-dependent kinases (CDKs) 4 and 6, driving retinoblastoma (RB) phosphorylation and progression through the G_1_ phase reviewed in [6]. Although CYCLIN D1 may have CDK-independent effects, nearly all CYCLIN D1 is found in complex with CDK4 in MCL cell lines and primary samples and highlights the importance of CDK4/6 in MCL transformation [7]. In addition to these genetic alterations, MCLs also exhibit dysregulation of other signaling pathways involved in cell proliferation and survival. To this end, loss of expression or inactivation of PTEN, a negative regulator of PI3 Kinase, have been reported in some MCLs, resulting in constitutive activation of AKT [8]. Importantly, casein kinase 2 (CK2), which also regulates the PTEN/PI3K/AKT pathway, is overexpressed in lymphoproliferative disorders, including MCL [9].

Similar to MCL, T-cell Acute Lymphoblastic Leukemia (T-ALL) is an aggressive hematologic malignancy resulting from the malignant transformation of T-cell progenitors. T-ALL accounts for 10-15% and 25% of pediatric and adult ALL cases, respectively reviewed in [10, 11]. PTEN inactivation and subsequent PI3K/AKT activation are common in this tumor type and are associated with CK2 mediated phosphorylation of the PTEN C-terminal tail [12]. CYCLIN D overexpression is also common in T-ALL, with the expression of particular CYCLIN D isoforms associated with distinct T-ALL subsets [13]. Importantly, the CYCLIN D3:CDK4/6 complex has been shown to have unique functions in the expansion of normally developing T cell progenitors and the induction of T cell leukemia [14, 15]. Furthermore, inhibition of CYCLIN D:CDK4/6 activity has been shown to abrogate proliferation in T-ALL cell lines and primary human cells as well as progression of disease in animal models of T-ALL [14, 15].

Owing to the near complete dysfunction of the RB pathway resulting from CYCLIN D over-expression, as well as CK2-mediated activation of the PI3K/AKT pro-survival pathway in the majority of MCLs and T-ALLs, we sought to determine whether dual targeting of these pathways might be an effective way by which to treat these malignancies. Towards this goal, we recently developed a dual inhibitor of CK2 and CDK4/6, ON108110, and examined its effects on the growth and apoptosis of MCL and T-ALL cell lines. X-ray crystallography studies combined with molecular modeling reveal that ON108110 binds to the active site of CK2 with high affinity and that it potently inhibits CK2 and CDK4/6 kinase activities. Furthermore, treatment of MCL and T-ALL cell lines with this compound effectively inhibits key signaling pathways mediated by these proteins, resulting in their apoptosis.

## RESULTS

### Derivation of a small molecule inhibitor of MCL and T-ALL cell growth

**Figure 1:**
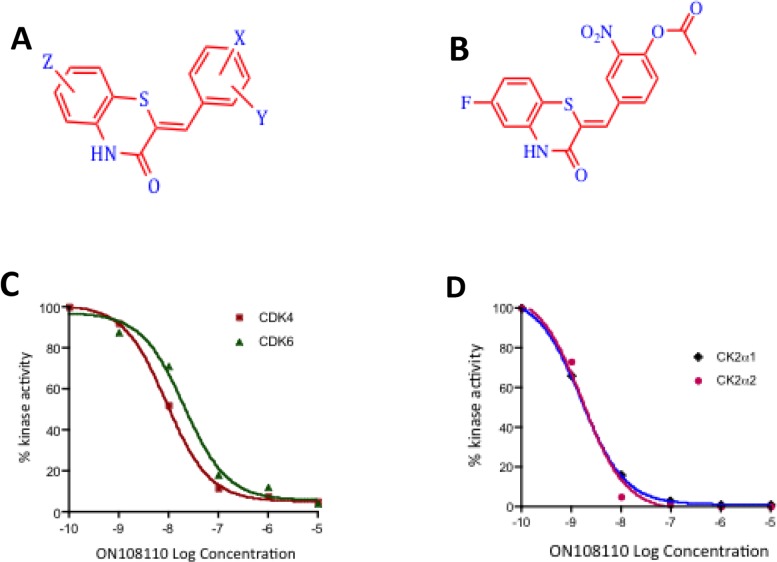
ON108110 is a multi-kinase inhibitor **(A)** Structure of the ON108XXO benzo thiazolidinnes chemotype. **(B)** Structure of ON108110. **(C)** Calculation of IC_50_ values against CDK4 and CDK6 and **(D)** CK2α1 and CK2α2 by ON108110. Recombinant proteins were incubated with the indicated concentrations of ON108110 and subjected to *in vitro* kinase assays as described in the materials and methods section. Values obtained were plotted as a function of log drug concentration. IC_50_ values were determined by performing sigmoidal non-linear regression with a variable slope.

As a part of this study, we developed a library of a novel ATP mimetic chemotype, the benzo thiazolidinnes, that contain a core thiazole structure and have synthesized approximately 300 compounds containing the basic backbone shown in Figure 1A. A primary screen aimed at determining the growth inhibitory (GI_50_) value against a panel of solid tumor and leukemic cell lines revealed that one of these compounds, ON108110 (Figure 1B), exhibited a 10-20-fold higher cytotoxicity against MCLs and T-ALLs compared to other tumor types (Table 1). Importantly and in contrast to its potent anti-cancer activity, ON108110 was found to exhibit little or no cytotoxicity towards normal human cells (Table 1). Subsequent testing of this compound for inhibitory activity against a panel of 285 functional kinases revealed that ON108110 is a multi-kinase inhibitor, with highest inhibitory activity against CDK4/6 and CK2 (Table 2), two kinases intimately associated with growth, survival and anti-apoptotic phenotypes of human tumor cells.

**Table 1:** Growth inhibitory activity of activity ON108110

Cell line	Tumor type	GI_50_ (μM)
Z138C	Mantle Cell Lymphoma	0.6
JVM2	Mantle Cell Lymphoma	0.3
GRANTA-519	Mantle Cell Lymphoma	0.15
HBL2	Mantle Cell Lymphoma	0.25
CEM	Acute Lymphoblastic Leukemia	0.18
MOLT-4	Acute Lymphoblastic Leukemia	0.5
RS-4-11	Acute Lymphoblastic Leukemia	0.24
LnCap	Prostate Cancer	>5.0
22Rv1	Prostate Cancer	>5.0
BT-20	Breast Cancer	2.5
MCF-7	Breast Cancer	0.8
MiaPaca-2	Pancreatic Cancer	2.6
AsPc1	Pancreatic Cancer	2.8
K562	Chronic Myeloid Leukemia	2.2
HEL	Erythroleukemia	0.9
hPBMC	Normal Human Peripharal Mononuclear Cells	>5.0

**Table 2:** Kinase inhibition profile of ON108110

Kinase	IC_50_ (nM)
CK2α1	3
CK2α2	2
CDK4/CyclinD1	11
CDK4/CyclinD3	15
CDK6/CyclinD1	2
CDK6/CyclinD3	24
CDK5/P25	7
PIM3	2

### Inhibition of CDK4/6 and CK2 kinase activity by ON108110

To confirm the that CDK4/6 and CK2 catalytic subunits were indeed the targets of ON108110, we independently tested its inhibitory activity in *in vitro* kinase assays using recombinant CDK4, CDK6, CK2α1 and CK2α2 (Figure 1). Our results showed that ON108110 is a potent inhibitor of both CDK4 and CDK6, with IC_50_ values of 6 and 10nM, respectively (Figure 1C). Palbociclib, an FDA-approved CDK4 inhibitor which showed similar inhibition at IC_50_ value of 6nM (data not shown) was used as a positive control. In addition to CDK4/6, ON108110 potently inhibited kinase activity of both CK2 catalytic subunits CK2α1 and CK2α2 with IC_50_ values of 3 and 2nM, respectively (Figure 1D). Silmitasertib (CX-4945), a commercially available CK2 inhibitor, inhibited these subunits with similar IC_50_ values (data not shown).

### ON108110 binds to protein kinase CK2

**Figure 2:**
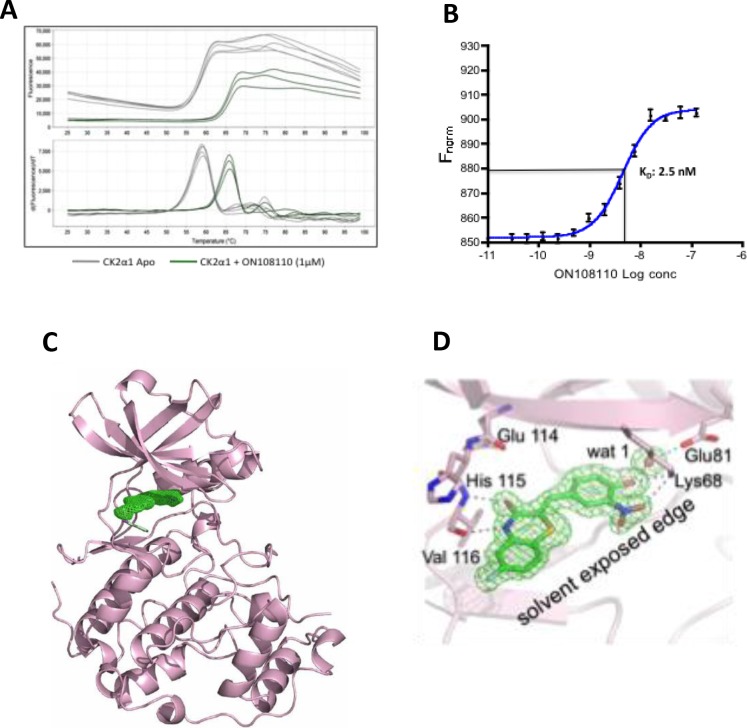
ON108110 binds to the active site pocket of CK2 **(A)** Unfolding of CK2α1 as monitored by DSF in the presence (apo protein) and absence of ON108110 (Figure 2a). **(B)** Dissociation constant of ON108110 in complex with CK2 as determined by MST. Binding curves were obtained using 16 concentrations of ON108110 (0.1 nM to 1,000nM). The K_d_ value was calculated using built-in curve fitting software (NanoTemper Technologies) and represents an average of 3 independent experiments. **(C)** 1.75 Å resolution structure of the CK2α1-ON 108110 co-crystal complex. ON 108110 (in green) occupies the ATP binding site of CK2α1. **(D)** ON108110 establishes hydrogen bonding with critical amino acids in the CK2α1 active site.

To determine the affinity and structural basis of ON108110-mediated inhibition and interaction with CK2, we performed differential scanning flourimetry (DSF) using recombinant CK2α1 kinase domain. While the CK2α1 apo protein showed a melting temperature (T_m_) of 58°C, incubation of this protein with ON108110 induced a 6.8°C shift in the T_m_ of CK2α1 to 64.8°C (Figure 2A), which is reflective of binding. We next determined the binding affinity constant of ON108110 to CK2α1 using microscale thermophoresis (MST) [16]. A highly purified preparation of recombinant CK2α1 was fluorescently labeled according to the instructions of the manufacturer and mixed with increasing concentrations of ON108110 (0.1nM-1000nM) and subjected to MST. The K_d_ values obtained from this analysis showed that ON108110 binds to CK2 with a K_d_ of 2.5nM (Figure 2B), demonstrating high affinity binding.

### X-ray Crystallographic Structure of the Nuclear Magnetic Resonance Analysis of the CK2α1-ON108110 interaction

To further understand the structural basis of CK2 inhibition, we performed crystallographic studies with CK2α1 in complex with ON108110. The co-crystal structure, which resolved at 1.75Å, revealed that ON108110 tightly binds in the CK2 active site pocket and that the compound established hydrogen bonding with the critical amino acids in the protein (Figure 2C). Interestingly, the O3 and N4 of the drug take the positions of the O6 and N1 groups of GMP-PNP, respectively, and maintain the same interactions with the backbone atoms of His115 and Val116. Moreover, the NO2 group of the drug is near the position that is occupied by the alpha phosphate of ATP or GTP and interacts by establishing a hydrogen bond with the side chain of Lys68 (Figure 2D).

### Prediction of ON108110 binding to the CDK6 kinase domain

**Figure 3:**
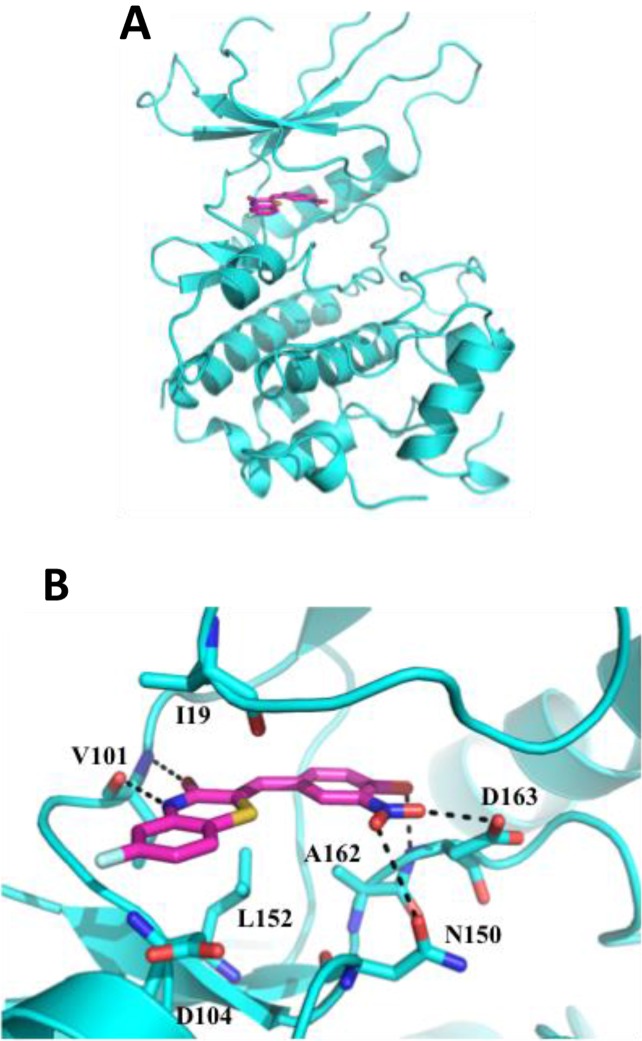
Molecular docking of CDK6-ON108110 ON108110 binding to CDK6 was predicted by docking and energy minimization using the X-ray crystal structure of CDK6-VCYCLIN and PD0332991 (2EUF) as a reference. Representations of the predicted lowest energy binding (CDK6/ON108110) were prepared using PyMOL. **(A)** Ribbon representation of the CDK6 kinase domain (cyan) bound to ON108110 (magenta). Small molecule is shown as stick. **(B)** Magnified view showing proximal residues of CDK6 to ON108110 predicted to be important for binding. Predicted hydrogen bonds are shown as dotted black lines.

To gain an understanding of the possible mode by which ON108110 binds to the ATP binding pocket of CDK4/6, molecular docking and energy minimization studies were conducted using the X-ray co-crystal structure of CDK6-VCYCLIN-PD0332991 as reference [17]. Binding of ON108110 to the CDK6 ATP binding site, like palbociclib, appears to be stabilized by various hydrogen bonds and multiple van der Waals interactions (Figure 3A). Specifically, two hydrogen bonds are predicted to form between the benzothiazinone N4 and O3 with the alpha chain of hinge residue Val101 (Figure 3B). Other hydrogen bonds are also predicted to form between the nitro and hydroxyl groups at positions 4 and 3 of the benzylidene moiety and Asn150 and of Asp163 (Figure 3B). Some of these hydrogen bonds are equivalent to those observed for palbociclib in the co-crystal structure. Overall, ON108110 is predicted to be binding tightly in the ATP binding site of CDK4/6, stabilized by numerous interactions, which explains the high affinity and inhibitory activity observed for this molecule towards CDK4/6.

### CK2 and CDK4/RB are overexpressed in MCL and T-ALL

**Figure 4:**
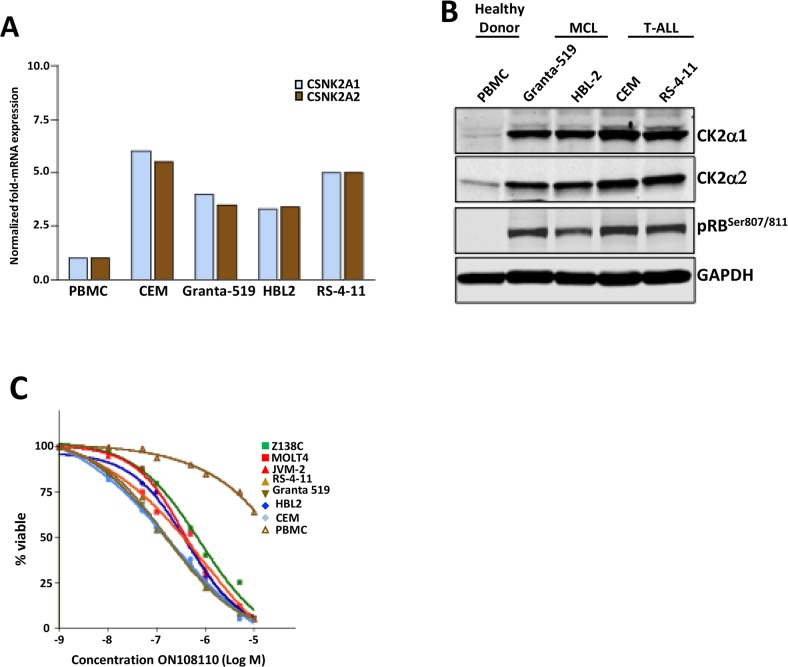
CK2 and CDK4 expression in cell lines and primary samples **(A)** mRNA levels of CK2 subunits in a panel of MCL and T-ALL leukemic cell lines and normal human PBMCs. **(B)** Protein levels of CK2 subunits and pRB^Ser807/811^ in a panel of MCL and T-ALL leukemic cell lines and normal human PBMCs. **(C)** Calculation of GI_50_ values against of ON108110 against MCL and ALL cell lines as well as normal PBMCs 72 hours post-treatment.

Based on the targets of ON108110 and the increased sensitivity of MCL and T-ALL cell lines to the anti-proliferative effects of this compound, we next examined the mRNA and protein levels of CK2 subunits and the phosphorylation status of target pRB in a panel of MCL and T-ALL cell lines. Figures 4A & 4B show that both MCL and T-ALL cell lines expressed significantly higher levels of *CKSN2A1* (CK2α1) and *CKSN2A2* (CK2α2) transcripts as well as protein subunits compared to normal peripheral blood mononuclear cells (PBMCs). In addition, western blot studies showed constitutive phosphorylation of pRB which is not seen in PBMCs. pRB and CK2 levels correlated with the sensitivity of these cell lines to the effects of ON108110 treatment as evidenced by their IC_50_ values (>100-fold more sensitive) when compared to that of PMBCs *in vitro* (Figure 4C).

### Effect of ON108110 on cell cycle progression and induction of apoptosis of MCL and T-ALL cells

**Figure 5:**
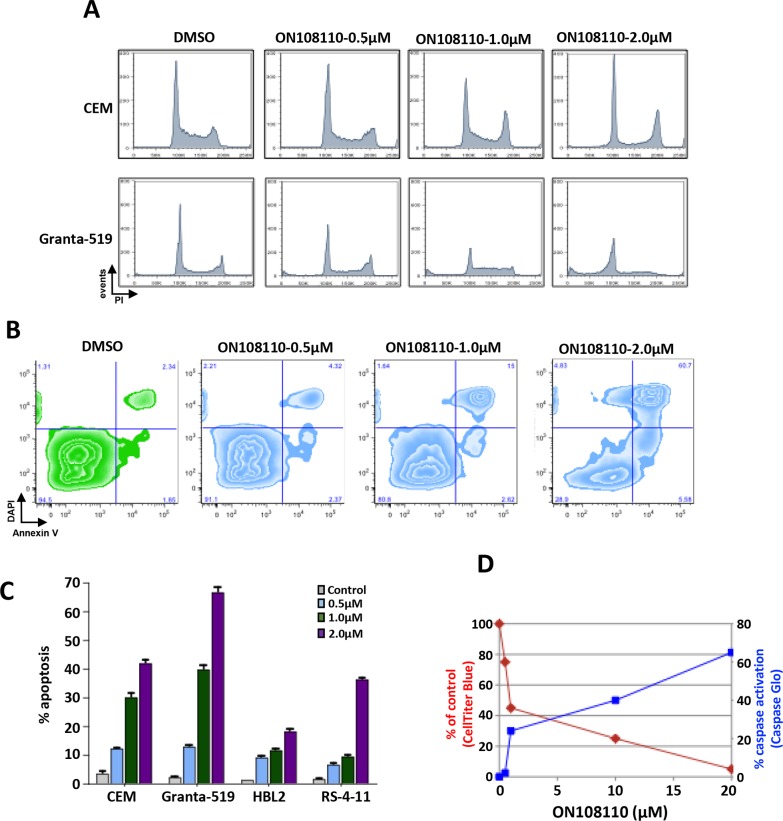
Modulation of cell cycle expression and induction of apoptosis in ON 108110-treated MCL and T-ALL cell lines **(A)** CEM and Granta-519 cells were treated with either DMSO or increasing concentrations of ON108110 (0.5-2.0μM) for 24 hours. Cell cycle analysis was performed using propidium iodide staining. **(B)** CEM, HBL-2, RS4-11 and Granta-519 cells were treated with either DMSO (vehicle) or increasing concentrations of ON108110 (0.5-2.0 μM) for 24 hours and stained with Annexin V/DAPI **(C)** Graphical representation of apoptosis induced as shown in B. **(D)** Granta-519 cells were treated with increasing concentrations of ON108110 for 24 hours. Caspase activation and viability were measured using the Caspase Glo 3/7 and Cell Titer Blue assay kits, respectively.

**Figure 6:**
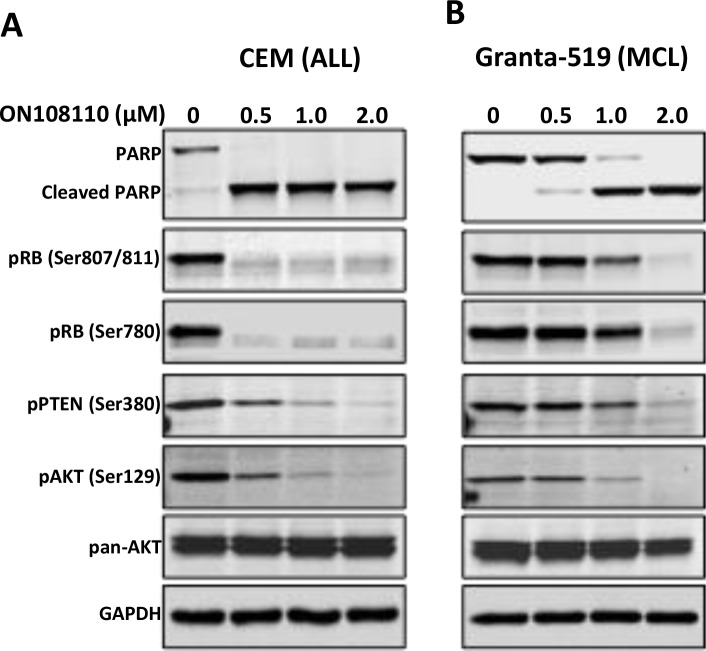
Inhibition of CK2/CDK4 mediated signaling by ON108110 **(A)** CEM and **(B)** Granta-519 cells were treated with increasing concentrations of ON 108110 for 24 hours. Whole cell extracts were subjected to western blot analysis using antibodies directed against the indicated proteins. GAPDH is shown as a loading control.

We next examined the effect of ON108110 treatment on cell cycle progression in MCL and T-ALL cell lines that are most sensitive to this compound, Granta-519 and CEM, respectively. For these studies, exponentially growing cells were treated with increasing concentrations of ON108110 for 24 hours. The cells were then harvested and subjected to propidium iodide staining and flow cytometric analysis. While there was no significant change in the profile of cells treated with DMSO over the course of the experiment, a rapid accumulation of the cells in the G_1_ phase of the cell cycle was evident following treatment with ON108110 (Figure 5A). However, at higher concentrations of the compound, a large proportion of the cells progressed through the S and G2/M phases and eventually began accumulating in the sub-G_1_ phase, suggesting induction of apoptosis at these concentrations (Figure 5A). To determine whether the sub-G_1_ phase cells correlated with the onset of apoptosis in cells treated with the ON108110, we analyzed the frequency of apoptotic cells using Annexin V staining. The results presented in (Figure 5B & 5C) show that while there is no evidence of activation of apoptosis DMSO treated cells, we observed a concentration-dependent increase in the frequency of apoptotic cells 24 hours post-treatment. Caspase activity and PARP cleavage also increased as a function of cell death in ON108110-treated cells (Figures 5D and 6). Together, these results suggest that dual CK2/CDK inhibition by ON108110 induced a potent cell cycle arrest in MCL and T-ALL cell lines that is accompanied by the induction apoptosis.

### ON108110 inhibits CK2 and CDK4/6 mediated oncogenic signaling in MCL and T-ALL cell lines

It has now been well established that the RB tumor suppressor is a primary target of CDK4 and CDK6 kinases. To determine the effects of ON108110 on the CDK/pRB signaling in MCL and T-ALL cell lines, Granta-519 and CEM cells were treated with increasing concentrations of ON108110 for 24 hours. Whole cell lysates were subjected to western blot analysis using antibodies against phosphorylated RB (Ser780 and Ser 807/811). The results of this study showed that while DMSO treated cells had high levels of phosphorylated RB, cells treated with ON108110 showed a concentration-dependent inhibition of CDK4/6-specific RB phosphorylation (Figure 6A & 6B).

CK2 has been shown to play an important role in hematopoietic malignancies, especially MCL and T-ALL [9, 18]. CK2 mediated phosphorylation and inactivation of tumor suppressor PTEN activates the PI3K/AKT signaling pathway, which is important in the survival of these and other tumor types. Because ON108110 inhibits CK2 kinase activity, we next examined phosphorylation status of the PTEN and AKT proteins in cells treated with this compound. As seen in Figure 6, ON108110-treated cells expressed lower levels of phospho-PTEN^Ser380^, a CK2 specific phosphorylation site that inactivates the PTEN phosphatase activity. Accordingly, we also detected a reduction in the level of AKT^Ser129^, which is phosphorylated by CK2 and promotes its activity [19]. These results provide a molecular basis for the observed induction of cell death in cells treated with ON108110.

## DISCUSSION

Aberrant cell cycle regulation is a common feature in most forms of cancer reviewed in [20]. In hematologic malignancies, including MCL and ALL, deregulated expression of D-type CYCLINS is frequently observed, which closely correlates with the high proliferative rate of these cells [4, 5]. Although there have been many efforts to develop selective inhibitors of CDK4/CDK6, initial studies with palbociclib, an FDA-approved selective small molecule inhibitor of CDK4 and CDK6 [21, 22], revealed that the drug was cytostatic when used as a monotherapy for MCL, resulting in disease stabilization with an 18% overall response rate [23]. In addition to CYCLIN D1, CK2 overexpression is a hallmark of T-ALL [18] and regulates multiple hematopoiesis-associated signaling cascades, including those involving PI3K and AKT, which are activated in MCL and T-ALL reviewed in [8]. In light of the key roles played by CYCLIN D1 and CK2 in hematological malignancies, it is not surprising that clinical trials aimed at testing small molecule inhibitors of these and other proteins in combination has gained increasing momentum.

In this communication, we examined the effects of ON108110, a dual inhibitor of CDK4/6 and protein kinase CK2 on the growth and viability of MCL and T-ALL. Our results show that ON108110 potently inhibits the kinase activities of these proteins *in vitro*. We also provide evidence demonstrating that targeting CK2 and CDK4/6-mediated pathways in MCL and T-ALL leads to potent cell cycle arrest and the induction of apoptosis. The G_1_ arrest seen with ON108110 treatment is clearly due to inhibition of phosphorylation of members of the RB family, a direct result of CDK4 and CDK6 inhibition. Furthermore, ON108110 acts as a potent inhibitor of CK2 mediated PI3K/AKT activation as measured by inhibition of CK2-specific phosphorylation sites of its substrates, PTEN^Ser380^ and AKT^Ser129^. Given the significance of the PTEN/PI3K/AKT signaling axis in T-ALL cell survival [18], our results provide a molecular basis for the observed induction of apoptosis in ON108110 treated cells and a strong rationale for targeting CK2 and CDK4/6 in MCL and T-ALL.

To gain insight into the structural basis of selectivity of ON108110 for CK2 and CDK6 we determined high-resolution crystal structures of the CK2–ON108110 complex. Structural studies showed that ON108110 binds tightly in the active site pocket of CK2α1. Furthermore. the co-crystal structure shows that ON108110 binds in the cleft between two domains that normally accommodate ATP. Importantly, ON108110 interacts with conserved amino acids and establishes hydrogen bonding with the surrounding amino acids and water molecule. Collectively, this extensive combination of direct and water-mediated hydrogen bonds and van der Waals contacts observed between ON108110 and CK2α establishes the structural basis for high affinity binding. Additionally, molecular docking and energy minimization studies with CDK6–ON108110 predict that ON108110 establishes various hydrogen bonds and multiple van der Waals interactions, which were similar to those observed with palbociclib. Collectively, ON108110 binds tightly in the ATP binding site of CDK6 and is stabilized by numerous interactions, which explains the potent inhibitory effect on CDK4/6.

In conclusion, our results indicate, that combined inhibition of CYCLIN D1/CDK4/RB and CK2/PI3K/AKT via a small molecule inhibitor of the CDK4/6 and CK2 kinases is effective in inducing cell death of MCL and T-ALL, particularly those that have aberrant upregulation of both signaling pathways.

## MATERIALS AND METHODS

### Cell lines

Cell lines were cultured in RPMI-1640 medium (Thermo Scientific) supplemented with 10% heat inactivated FBS and penicillin-streptomycin at 37°C under humidified conditions and 5% CO_2_. All cell lines tested negative for mycoplasma. To determine GI_50_ values, cells were seeded at a density of 2.5×10^3^ cells-0.1ml^−1^ per well of a 96-well plate. Compounds were added 24 hrs post-plating at the indicated concentrations. Cell counts were determined from duplicate wells 96 hrs post-treatment using the Cell Titer Blue assay in conjunction with the GloMax plate reader (Promega).

Peripheral blood samples were obtained from patients at the Icahn School of Medicine at Mount Sinai with informed and written consent for tissue banking following a protocol approved by the Institutional Review Board (MSSM IRB#11-1669) and in accordance with the Declaration of Helsinki. Peripheral blood mononuclear cells were pelleted by low-speed centrifugation, resuspended in media composed of 90% fetal bovine serum (FBS) and 10% DMSO (Sigma), frozen slowly in the vapor phase of liquid nitrogen in multiple cryovials, and stored in liquid nitrogen.

### Reagents and antibodies

ON108110 was obtained from Onconova Therapeutics, Inc. PD-0332991 and CX-4945 were purchased from Selleckchem. Antibodies directed against PARP (catalog #9542), Caspase 3 (catalog #9665), phospho-RB^Ser780^ (catalog #9307), phospho RB^S807/811^(catalog #9308), RB (catalog #9309), phospho-Akt^Ser129^ (catalog #2974), were obtained from Cell Signaling Technologies. GAPDH (catalog #SC-47724), AKT (catalog #SC-1618), CYCLIN D1 (catalog #SC-8396) and p21-specific (catalog #SC-756) antisera were purchased from Santa Cruz Biotechnology.

### Kinase assays

Initial testing of ON108110 against a panel of 285 functional kinases was performed by Reaction Biology Corp (Malvern, PA). Recombinant CDK4/CYCLIN D1 or CK2α1 were mixed with their respective synthetic peptide substrates (10ng) and incubated with DMSO or increasing concentrations of ON108110 in 20μl kinase buffer [50mM Tris-HCl (pH 7.5), 10mM MgCl_2_, 1mM EGTA, 2mM DTT and 0.01% NP-40] for 30 min. at room temperature. Kinase reactions were carried out for 20 min at 30°C in the presence of 5μl of substrate-ATP mixture (1μg RB, 20μM ATP, 20μCi γ-^32^P-ATP). The reactions were incubated at 30°C for 20 minutes, terminated by addition of 0.5M O-phosphoric acid and 10μl of the reaction mixture was spotted on P-30 Filtermat membranes (Wallac #1450-523). The filtermat was air-dried, blocked with methanol for 2 minutes followed by three washes with 75mM phosphoric acid. The filtermat was then air-dried and analyzed using a phosphoimager. Densitometric values were plotted as a function of log drug concentration using Prism 4 GraphPad software and the IC_50_ values determined by plotting sigmoidal non-linear regression curves with variable slope (n=3).

### RNA expression in MCL and T-ALL cell lines

For quantitative RT-PCR (qRT-PCR), total RNA was extracted using the RNeasy mini kit (Qiagen) and subjected to reverse transcription using Superscript III (Life Technologies). Q-PCR of the resulting cDNA templates was performed using Power SYBR Green Master Mix (Applied Biosystems). Expression levels of individual genes were normalized to ß-actin. Amplification of *CKSN2A1* (CK2α1), *CKSN2A1* (CK2α2), and β-actin was performed using Applied Biosystems proprietary primer-probe sets.

### Western blot analysis

Cells were grown as indicated, lysed in lysis buffer (20mM Tris, pH 8.0/ 137mM NaCl/ 10% glycerol/ 1% NP-40) supplemented with 1X protease and phosphatase inhibitor cocktail on ice and resolved by SDS-PAGE. The proteins were transferred to a nitrocellulose membrane and subjected to Western blot analysis using the indicated antibodies. Proteins were visualized using an Odyssey imaging system (LI-COR Biosciences).

### Flow cytometric analysis

To determine cell cycle distribution, 70% ethanol fixed cells were washed with phosphate buffered saline (PBS), resuspended in PBS and incubated with RNase and propidium iodide at 37°C for 20 min. DNA content was analyzed on a FACS Calibur (BD Immunocytometry Systems) and the data analyzed using Flowjo software (Treestar). Annexin V staining was measured using the FITC Annexin V Apoptosis Detection Kit I (BD Biosciences) according to the instructions of the manufacturer except that DAPI was substituted at a concentration of 1mg ml^−1^ to measure DNA content.

### Caspase activation assay

Caspase-3/7 activation was measured using the Caspase-Glo^®^ 3/7 kit as recommended by the manufacturer (Promega).

### CK2α1 protein expression and purification

Amino acid residues 3-335 of CK2α1 were cloned using BamH1 and HindIII into pET28bsmt3-SUMO fusion vector, which encodes a N-Terminal His6 Tag followed by a SUMO (small ubiquitin-related modifier) protein and SUMO protease cleavage site. The nucleotide sequence was confirmed by DNA sequencing. CK2α1 was expressed in Escherichia coli BL21-DE3 (RIL), cells and lysed by sonication. Cell lysate was clarified by centrifugation at 18,000 rpm for 1 hour at 4°C. The supernatant was filtered and loaded onto a Ni- NTA column equilibrated with 25mM Tris pH-8.5, 0.5M NaCl, 20mM imidazole pH 8.0, 86 10% Glycerol and 2mM β-mercaptoethanol. The protein was eluted using elution buffer containing 25mM Tris pH8.5, 0.3M NaCl, 250mM imidazole pH 8.0 and 2mM β- mercaptoethanol. Fractions containing CK2α1 were pooled and dialyzed overnight against 25mM Tris, pH8.5, 0.3M NaCl and 2mM β-mercaptoethanol. The SUMO tag was cleaved by adding ULP1 protease (5mg ml-1) to the dialysis buffer. Cleaved CK2α1 was then passed over a second Ni2+-NTA column to collect CK2α1 and remove the His-Tag SUMO and His Tag-ULP1 protease. The flowthrough was collected, concentrated, passed through a Heparin Column and further purified using size exclusion chromatography using a Superdex 75 gel filtration column. Fractions with >95% pure CK2α1 (as monitored by SDS-PAGE electrophoresis) were collected and concentrated using an Amicon centrifugal concentrator. The purified protein was quantified aliquots were snap frozen and stored at −80°C.

### CK2α1-ON108600 co-crystallization

2μl of 10 mg ml-1 purified CK2α1 protein solution was mixed with an equal volume of reservoir solution (21% PEG-5000, 0.2M ammonium sulfate, 0.1M Tris HCl and 10 mM AMP-PNP). Thin, needle-like crystals were obtained by the vapor diffusion method at 18°C. Crystals obtained from microseeding were soaked with ON108110 dissolved in DMSO (1mM). Co-crystallization was also performed by the hanging drop vapor diffusion method at 18°C. Crystals appeared over a period of 7 days. For data collection, the crystals were cryoprotected by stepwise soaking for 5 minutes in mother liquor solutions containing 5–30% (v/v) glycerol and subsequently flash frozen in liquid nitrogen. X-ray diffraction data were collected on beamline X25 at Brookhaven National Laboratory. The crystals diffracted to a 1.75-Å resolution with synchrotron radiation at the platinum LIII absorption edge (λ = 1.0722 Å). The crystals belonged to the space group P21.

### Molecular docking of ON108110- CDK6

ON108110 small molecule was generated using SKETCHER (CCP4 suite) and superimposed in COOT to the ligand of the X-ray co-crystal structure of CDK6-Vcyclin-PD0332991 (2EUF). Binding positions of ON108110 were analyzed using restrained docking in FireDock and the predicted lowest energy binding was visualized and graphed using PyMOL.

### Differential scanning fluorimetry

1μM of in-house purified recombinant CK2α1 was diluted in protein buffer and added to a final concentration of 1X of Sypro Orange Dye in the presence of DMSO or increasing concentrations of ON108110. Following a 20 min incubation at room temperature, 20μl samples were pipetted into a clear MicroAmp Fast 96-well Reaction PCR plates and sealed with MicroAmp Optical Adhesive Film. The assay was performed using a StepOne Plus RT-PCR instrument (Applied Biosystems) using an increasing temperature gradient of 20 to 90°C in 0.2°C increments using a 200- millisecond stabilization delay. Florescence readings were acquired with excitation and emission wavelengths of 580±10 nm and 624±14 nm respectively. A customized analysis program (Step One Plus Protein Thermal Shift Software) was used to calculate the T_m_ from each fluorescence profile (Boltzmann method) and the T_m_ of the fluorescence (derivative method). Derivative T_m_ values were obtained from the top of the peak in the derivative plot, while the Boltzmann T_m_ values calculated from the inflection point of the fluorescence plot.

### Microscale thermophoresis

Purified His6-CK2α1 (kinase domain) was labeled with red NT-647 cysteine-reactive dye according to the manufacturer (NanoTemper Technologies). A fixed quantity of NT-647-labeled CK2α1 (50nM) was incubated with the indicated concentrations of ON108110 in binding buffer (20 mM Tris-HCl, pH 7.5, 150 mM NaCl, 1 mM MgCl2, 0.1% Triton X-100, and 2% DMSO) at room temperature in the dark for 30 min. Samples were then loaded into hydrophilic capillaries (NanoTemper Technologies) and binding was measured using the Monolith NT.115 (NanoTemper Technologies). Binding data were analyzed using thermophoresis or hot/cold analysis, as previously described [24] prior to normalization to either ΔF_norm_[%(10^*^(F_norm_(bound)-F_norm_(unbound)) or fraction bound (ΔF_norm_[%]/amplitude) [24]. Dissociation constants (K_d_) were calculated using built-in curve-fitting software provided by the manufacturer (NanoTemper Technologies).

## Abbreviations

CK2: casein kinase 2
CDK4/6: cyclin-dependent kinase 4/6
RB: retinoblastoma
MCL: mantle cell lymphoma
T-ALL: T-cell acute lymphocytic leuekemia
MST: microscale thermophoresis
DSF: differential scanning fluorimetry
PI3K: phosphatidylinositol 3-kinase
PTEN: phosphatase and tensin homolog
AKT: protein kinase B
PBMC: peripheral blood mononuclear cell
